# Robotic Repair of a Paraesophageal Hernia After an Open Nissen Fundoplication: Case Presentation and Clinical Discussion

**DOI:** 10.7759/cureus.64757

**Published:** 2024-07-17

**Authors:** Alyssa M Hammar, Leanna Zelle, Erin Nischwitz, Andrew A Wheeler, Milot Thaqi

**Affiliations:** 1 Department of General Surgery, University of Missouri School of Medicine, Columbia, USA

**Keywords:** paraesophageal hernia, intra-abdominal adhesions, unplanned reoperation, cameron ulcer, recurrent hernia, robotic hiatal hernia, paraesophageal hiatal hernia, dysphagia surgery, gastroesophageal reflux disease (gerd), recurrent gi bleeding

## Abstract

We present a female in her sixties with a recurrent paraesophageal hernia status post open Nissen fundoplication and multiple esophageal dilations who underwent a robotic paraesophageal hernia repair, with extensive lysis of adhesions. The stomach and esophagus were dissected off the crura and the previous wrap was undone. Once the entirety of the stomach and esophagus were freed from their surrounding structures, the hernia sac was able to be excised. The crural defect was closed and gastropexy was performed. The patient had an uneventful postoperative course and was discharged home. This case is presented to provide evidence that robotic repair presents a viable option in the reoperation of patients following an open Nissen fundoplication as well as provide an overview of the types of hiatal hernias and the indications and options for surgical intervention.

## Introduction

Paraesophageal hernias comprise approximately five percent of hiatal hernias. Surgical management is indicated when a patient becomes symptomatic and is refractory to medical management [1]. With the advancement of robotic and laparoscopic surgery, open paraoesophageal hernia repair with Nissen fundoplication has decreased in popularity and poses increased difficulty if future intervention is indicated due to the degree of scar tissue and adhesions. Recurrence of paraesophageal hernias is not an uncommon occurrence. Current literature supports a high recurrence rate, ranging from 25 to 42 percent after more than five years following a hiatal hernia crural repair with either a Nissen or Toupe fundoplication [2]. Studies estimate that approximately 15% will require re-operation, these reoperations carry a higher morbidity (15-40%) and mortality due in part to the presence of adhesions and distorted anatomy [3]. Therefore, when selecting a secondary operation, the selection of the most appropriate approach is critical. Here we will present a case of a female in her sixties with a symptomatic recurrent paraesophageal hernia following an open trans-abdominal paraesophageal hernia repair with a Nissen fundoplication and multiple esophageal dilations.

Esophagogastroduodenoscopy (EGD) revealed a recurrent hernia with Cameron ulcers. The patient subsequently underwent laparoscopic paraesophageal hernia repair (robotic-assisted), gastropexy, and gastrorrhaphy. This case was previously presented as an e-video at the 2024 SAGES Meeting on April 20, 2024. 

## Case presentation

An approximately 65-year-old female presented to the emergency department with a chief complaint of left-sided chest pain and shortness of breath for two weeks in duration. Her past medical history was significant for chronic obstructive pulmonary disease, hypothyroidism, and a history of dysphagia and esophageal dysmotility status post open, trans-abdominal paraesophageal hernia repair with a Nissen fundoplication eight years prior. She additionally had undergone multiple EGDs with dilations to aid with her dysphagia following her hernia repair. Her body mass index was 26.6. The remainder of her past medical history is listed in Table 1. She was noted to have had an upper endoscopy two months prior with evidence of Cameron ulcers and a recurrence of her paraesophageal hernia. On presentation, the patient reported non-compliance with her proton pump inhibitor and reported only taking occasional over-the-counter calcium carbonate tablets to assist with her gastroesophageal reflux (GERD) symptoms. She denied any evidence of hematochezia or melena. 

**Table 1:** Past Medical and Surgical History

History Type	Patient History
Past Medical	Chronic obstructive pulmonary disease, fibromyalgia, hypothyroidism, esophageal dysmotility and stricture
Past Surgical	Open paraesophageal hernia repair with Nissen fundoplication, hysterectomy, laparoscopic appendectomy, laparoscopic cholecystectomy, and multiple esophagogastroduodenoscopies with dilation
Medications	Percocet, albuterol, aspirin, atorvastatin, desvenlafaxine, levothyroxine, mirtazapine, olanzapine, and tiotropium

**Figure 1:**
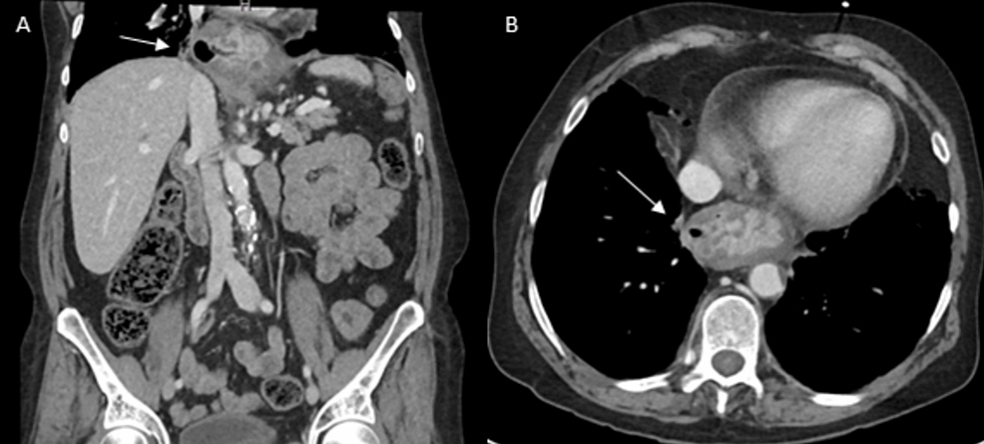
CT Abdomen and Pelvis The image on the left (A) is a coronal CT with intravenous contrast and the image on the right (B) is an axial view of the same CT study. In both images, the arrows point to the stomach that has herniated into the thoracic cavity through the crura of the diaphragm.

On presentation to the emergency room, the patient was noted to be tachycardic with a heart rate in the 110’s, blood pressure within normal limits, and appropriate oxygen saturation. The patient underwent a complete blood count (CBC) which was notable for a hemoglobin of 5.3. The remainder of her relevant lab findings are listed in Table 2. Two large bore intravenous (IV) catheters were placed, and the patient was administered three units of packed red blood cells (PRBCs). A computed tomography (CT) scan of the abdomen and pelvis (Figure 1) was obtained in the emergency department which revealed no acute processes but demonstrated a moderate hiatal hernia with mucosal thickening along the gastroesophageal (GE) junction, consistent with chronic gastritis. Following administration of the units of PRBCs, the patient's hemoglobin responded appropriately, and her heart rate improved to within normal limits. She was admitted to the hospital and scheduled for an EGD the next day. 

**Table 2:** Initial Lab Values on Presentation

Laboratory Type	Patient Value	Reference Value
White Blood Cell	7.59 x 10(9)/L	3.5-10.50 x 10(9)/L
Hemoglobin	5.3 g/dL	12.0-15.5 g/dL
Hematocrit	18.7 %	34.9-44.5 %
Platelet	264 x 10(9)/L	150-450 x 10(9)/L
Sodium	136 mmol/L	126-145 mmol/L
Potassium	4.0 mmol/L	3.5-5.1 mmol/L
Creatinine	.82 mg/dL	.50-1.00 mg/dL
Albumin	4.1 g/dL	3.5-5.2 g/dL

**Figure 2:**
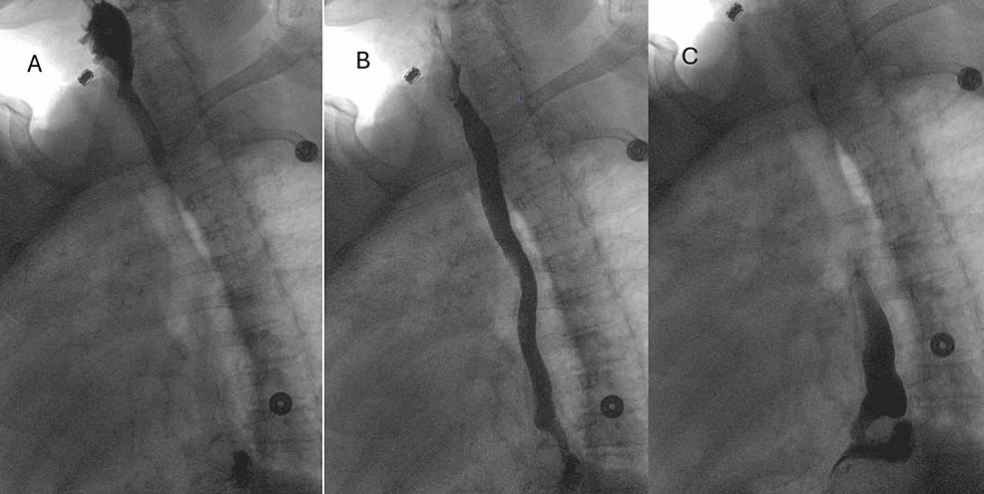
Upper Gastrointestinal Series Upper gastrointestinal (GI) series revealing a moderate paraesophageal hernia containing the gastric fundus (A), a questionable pulsion diverticulum, mild esophageal dysmotility (B), and mild gastroesophageal reflux (C).

An EGD was performed and revealed a 10cm hiatal hernia with a Cameron ulcer and mild erythema along the stomach antrum. She was started on an IV infusion of a proton pump inhibitor and oral sucralfate and general surgery was consulted. General surgery ordered an upper gastrointestinal series (Figure 2) to evaluate her motility and scheduled her for operative intervention during the same admission. 

**Figure 3:**
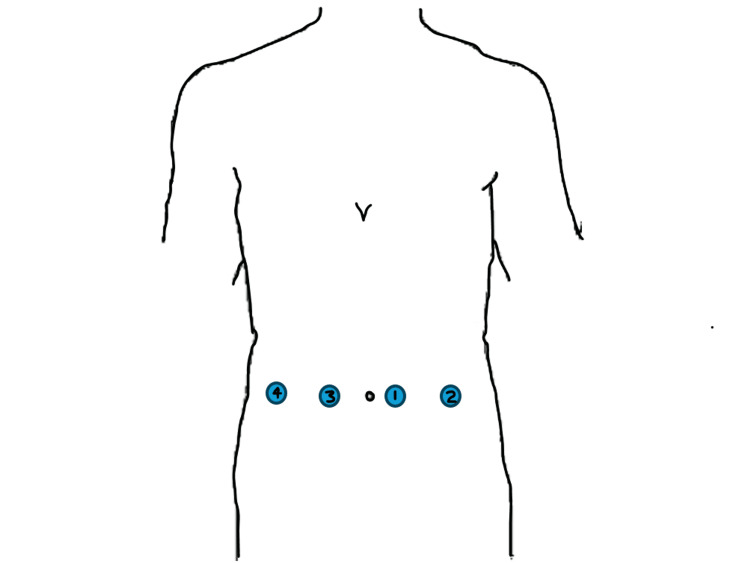
Port Placements Four 8mm robotic ports were placed in a linear fashion across her abdomen, approximately 15cm inferior to the xiphoid under direct visualization. The ports were placed in the numerical order as shown in the figure.

The patient underwent a robot-assisted laparoscopic repair of the paraesophageal hernia during the same admission. The patient was positioned in the supine position and following administration of general endotracheal intubation, and standard sterile draping, four 8mm robotic ports were placed in a linear fashion across her abdomen, approximately 15cm inferior to the xiphoid under direct vision following dissection of noted adhesions within the area (Figure 3).

**Figure 4:**
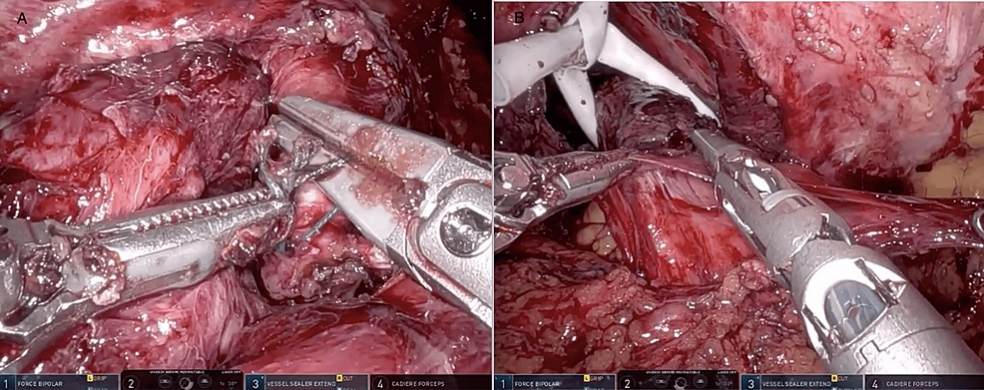
Intraoperative Images of Previous Hernia Repair Takedown and Hernia Sac Dissection The image shows the removal of the suture holding together the previous hiatal hernia repair (A) as well as the dissection of the hernia sac (B).

Adhesiolysis was then performed with the robot for greater than one hour until the stomach was visualized. The liver was adherent to the abdominal wall therefore a retractor was not needed to facilitate exposure. Several adhesions were encountered at the level of the hiatus which were taken down with scissors to expose the right crus. Dissection was then carried circumferentially along the stomach. During the dissection, a prior stitch at the hiatus posteriorly was encountered holding the previous Nissen Fundoplication wrap in place. This was ligated and removed, upon removal a small amount of gastric content leaked from the stomach posteriorly, with a noted gastrotomy in the area. After additional dissection of the stomach off the hiatus, the left crus was identified. Dissection was continued to separate the esophagus off the aorta, carefully cauterizing small branching aortic vessels supplying the distal esophagus. The previous hernia repair and Nissen wrap were undone (Figure 4). The stomach was then placed in a more anatomical position and a Penrose drain was placed around the esophagus to aid with retraction. The hernia sac was dissected off its attachments in the right thoracic cavity, and once it was freed from the pleural space, the sac was excised. 

With the aid of the Penrose, dissection was continued to the level of the pulmonary veins taking care to preserve the left and right vagus nerves. Once the dissection was sufficient to ensure at least 3cm of esophagus present within the abdomen, crural closure was performed. A 0-permanent barbed suture was used to repair the crural defect in a running fashion. 

**Figure 5:**
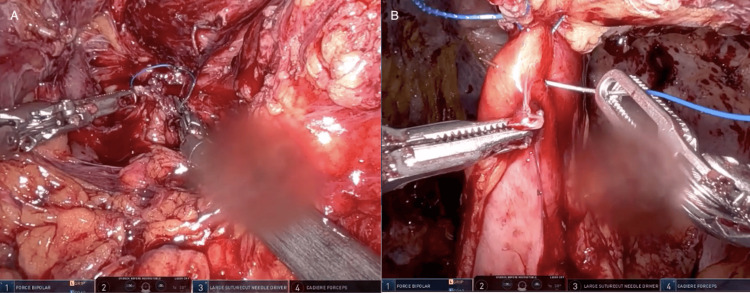
Intraoperative Images of Hiatal Closure and Gastropexy The figure in the left shows the closure of the crural defect with a 0-permanent barbed suture in a running fashion (A). The image on the right displays the linear gastropexy being performed using a 2-0 permanent barbed suture, starting proximally on the stomach body, and suturing to the abdominal wall in a running fashion (B).

Following evaluation of the stomach, the gastrotomy from the prior crural stitch was noted along the greater curve of the stomach on the posterior aspect, just distal to the GE junction. This was closed primarily in two layers using 3-0 barbed non-permanent suture in a running fashion. An EGD was then performed to ensure appropriate closure of the gastrotomy and to evaluate the viability of the remainder of the stomach. The pylorus appeared to be widely patent, the stomach mucosa appeared to be healthy and on retroflection of the scope, the gastrorrhaphy appeared to be repaired appropriately. The remainder of the stomach and esophagus were evaluated and did not reveal any areas of ischemia. A linear gastropexy was then performed using a 2-0 permanent barbed suture, starting proximally on the stomach body, and suturing to the abdominal wall in a running fashion (Figure 5). The hernia sac was then removed from the abdomen and this concluded the procedure. The patient was awakened from anesthesia and extubated. 

The patient was taken to the post-anesthesia care unit in stable condition and returned to the floor under the care of the surgical team. The patient had an uneventful postoperative course. On postoperative day (POD) one, an upper GI series was performed which revealed no evidence of a leak and she was started on a clear liquid diet, on POD two, the patient was tolerating liquids and had return of bowel function, and on POD three, the patient advanced to soft mechanical diet, with significant resolution of her abdominal pain. The patient was subsequently discharged home on POD four. 

## Discussion

Hiatal hernias are classified into four subtypes. Type I hernias, also referred to as sliding hernias, make up over 90% of cases and are characterized by ascent of the GE junction through the crus of the diaphragm while the fundus remains below the GE junction. Type I hernias are associated with increased rates of GERD, esophagitis, and Barrett's esophagus compared to the general population but are more frequently asymptomatic than type II-IV hiatal hernias [4]. Type II-IV hernias are all classified as paraesophageal hernias. Type II hernias, known as pure paraesophageal hernias, are characterized by the fundus of the stomach ascending through the diaphragmatic hiatus adjacent to the esophagus, while the GE junction remains in its normal anatomical position. Type III hernias are characterized by both the fundus and the GE junction ascending through the diaphragmatic hiatus. Type III hernias are often thought of as a combination of type I and II hernias. Type IV hernias are characterized by a structure other than the stomach ascending through the hiatus, including small bowel, colon, omentum, peritoneum, or spleen within the hernia sac. Diagnostic workup to differentiate between the types of hernia included barium swallow radiography, EGD, esophageal manometry, pH testing, or computed tomography [4]. 

The treatment approach for hiatal hernias ranges from medical to surgical. Historically, elective surgery was recommended for all hiatal hernias. However, the current SAGES guidelines have been updated to recommend surgical repair for symptomatic hernias only [1]. Non-surgical intervention is recommended for asymptomatic or minimally symptomatic type I hernias and includes lifestyle changes or medications. Examples of lifestyle changes that have been proven to be beneficial for symptomatic hiatal hernias include weight loss, elevation of the head of the bed by eight inches, avoiding foods that trigger gastroesophageal reflux, alcohol cessation, and avoiding ingestion of meals two to three hours before bed. Medications that can be useful include PPIs, histamine blockers, and over-the-counter antacids [4]. Symptomology for hiatal hernias varies widely depending on the type of hernia and the patient’s anatomy. Symptoms commonly include heartburn, early satiety, chest pain, dyspnea, dysphagia, and regurgitation [1]. Patients often have difficulty determining if they are asymptomatic, as the onset of these symptoms is insidious, non-specific, and easily attributed to other causes. Thus, in cases that are mild to moderately symptomatic, the determination of a patient’s treatment approach is made by considering the patient’s age, comorbidities, response to medications, and personal health goals. 

When lifestyle changes and medications fail for type I hiatal hernias, surgery is indicated. Compared to type I hiatal hernias, type II-IV hernias have a higher side effect profile, are less likely to respond to non-surgical treatments, and have a higher risk of life-threatening complications like obstructions or volvulus. Therefore, surgical treatment is the only definitive treatment for type II-IV hernias [4]. Elective hernia repairs are preferred, and SAGES recommends performing bariatric surgery simultaneously with hiatal hernia repair surgery in patients who would benefit from both interventions. If a patient presents with an acute, life-threatening complication like obstruction or volvulus, immediate surgery is indicated and may require reduction of the stomach with limited resection [5]. 

When surgical management is deemed appropriate, there are multiple methods commonly used, including open abdominal, open thoracic, laparoscopic, and laparoscopic with robotic assistance. Laparoscopic paraesophageal hernia repair is associated with a significant reduction in the length of hospital stay and with a decreased risk of mortality regardless of age as compared to either abdominal or thoracic open repair. Laparoscopic surgery holds a mortality risk of 0.57%, whereas open abdominal and thoracic surgeries carry a risk of 1.33% and 1.24% respectively [6]. Open thoracic is associated with the longest hospital stay and increased risk of postoperative mechanical ventilation and pulmonary embolism. However, open repair was traditionally associated with lower recurrence rates. This is an important consideration given the greater morbidity and mortality risk of recurrent surgeries [3]. Recent evidence suggests that while this may have previously been true, recurrence rates reach similar numbers as laparoscopic technique has evolved and as surgeons become equally experienced in laparoscopic repair [7]. With these considerations, laparoscopic abdominal repair with or without mesh placement is considered the standard of care in an uncomplicated paraesophageal hernia repair [1]. 

Though recurrence of the paraesophageal hernia is an infrequent outcome, when recurrence occurs it is not consistently symptomatic, and even less frequently these recurrences require repeat surgical intervention. Current literature indicates that anywhere from 5 to 59% of repairs will result in recurrence. However, only 15% of those will go on to require repeat surgical intervention [3]. The risk for recurrence is primarily determined by the size of the hernia repaired [8]. The decision to re-operate is dependent on radiologic evidence and the severity of symptoms. In those with minimal evidence of recurrence on radiology and minimal symptoms, medical management as discussed above is appropriate. This is true for most patients with recurrence. In those with mild to moderate radiologic recurrence with significant symptoms and those with severe radiologic recurrence, even if with minimal symptoms, surgical intervention should be considered. 

When planning for recurrent treatment, one must first select an appropriate approach, this decision is influenced by the approach of their previous operation, whether they have already had reoperations, and the level of concern for complications. As with the index repair, laparoscopy is preferable if considered feasible. Careful dissection of the hernia sac from surrounding structures is considered likely to reduce future herniation. As is excision, but the evidence is less overwhelming. If sac excision would present a significant risk of complication, it may be avoided [5]. The use of mesh is also somewhat controversial, as it is debatably associated with a lower recurrence rate and is associated with a significant potential side effect profile. Prosthetic mesh may become infected requiring surgical removal and has also been seen to erode into the esophagus or gastric cardia or cause esophageal stenosis. In large hiatal hernias, it is associated with decreased recurrence rates short-term, but long-term data is not currently robust. Some research shows that vertical mesh strips may provide the benefit of mesh reinforcement while also lowering these risks [8]. At this point, there is not adequate evidence to support the use of mesh in all repairs as complications can be severe. 

Fundoplication has previously been considered a routine step in the repair of paraesophageal hernias. Nissen fundoplication provides the greatest reflux control, but this selection may worsen dysmotility and should be avoided in patient populations with preexisting dysmotility. Partial fundoplication is associated with a lower risk of postoperative dysphagia but does not provide as robust reflux control. Toupet, or posterior partial, fundoplication, would be the more common choice, especially if there is significant concern for dysmotility. Dor, or anterior partial, fundoplication is a less commonly selected approach and is typically used when esophageal myotomy is also indicated [3]. However, recent evidence indicates that it is highly associated with dysphagia, and for those with less optimal reflux control, PPIs were adequate to control symptoms [9]. Therefore, routine fundoplication is not indicated. Gastropexy is considered a safe addition to hernia repair. It is especially helpful in cases where there is significant concern for torsion. There is also evidence to suggest that it may lower rates of recurrence. One prospective study indicated that of all patients followed for a year, there were no cases of recurrence. A small portion of the study additionally completed a two-year follow-up, also without incidence of recurrence [10]. As it is generally considered safe and provides a potentially large decrease in recurrence, it is a very reasonable addition to a paraesophageal hernia repair. 

Recurrence after repeat paraesophageal hernia repair is determined by similar risk factors as was the original repair, larger hernias being most likely to recur. Success rates of revisional surgery are similar to those of primary repair [5]. Long-term improvement of gastroesophageal reflux disease leads to high levels of patient satisfaction and improved quality of life scores. In an investigation of long-term outcomes of paraesophageal repair, patients experience long-term symptom resolution and satisfaction. The most frequent complaints were early satiety, nausea, and pain with swallowing. Even with small anatomic recurrences, patients tend to have good symptom control [11]. Studies do not currently recommend routine radiographic monitoring after surgery as most recurrences are not clinically significant [5]. 

## Conclusions

Recurrent paraesophageal hernias following open Nissen fundoplication are a rare encounter. The case is challenging due to the adhesions following an open repair. Nevertheless, despite the extensive adhesiolysis and complex dissection, laparoscopic repair with robotic assistance presents a viable option in the reoperation of these patients.
